# Identification of *Fusarium verticillioides* Isolates and Their Impact on Seed Germination and Biochemical Profiles in Maize

**DOI:** 10.1002/pei3.70104

**Published:** 2025-12-23

**Authors:** Abdul Rashid Hudu, Fredrick Kankam, Ibrahim Abdul‐Rahman Nanmang, Mohammed Mujitaba Dawuda, Hamdala Hunsulu Hamid, Nelson Opoku

**Affiliations:** ^1^ Department of Agricultural Biotechnology, Faculty of Agriculture, Food and Consumer Sciences University for Development Studies Nyankpala Ghana; ^2^ Department of Horticulture, Faculty of Agriculture, Food and Consumer Sciences University for Development Studies Nyankpala Ghana; ^3^ Department of Biotechnology and Molecular Biology, Faculty of Biosciences University for Development Studies Nyankpala Ghana

**Keywords:** antioxidant, *Fusarium verticillioides*, germination, hydrogen peroxide, maize seedling, malondialdehyde

## Abstract

*Fusarium verticillioides* is a common fungal pathogen of maize that causes significant losses in seed quality and seedling performance. Despite the high prevalence of this fungus in Ghanaian maize varieties, there is relatively little knowledge of the impact of *F. verticillioides* on seedling performance of Ghanaian maize varieties. The aim of this study was to isolate *F. verticillioides* isolates in the Bihilifa maize variety and to evaluate the effects of these isolates on germination‐ and biochemical‐linked traits. Six fungal cultures were isolated from the maize seeds. Based on phenotypic and phylogenetic analysis using translation elongation factor 1 alpha (TEF1‐α) gene, three isolates were identified as *F. verticillioides*: Fv‐B12024, Fv‐B22024, and Fv‐B32024, and were used in the germination and biochemical assay. All three isolates significantly reduced the germination‐linked traits: germination percent (GP), root length (RL), shoot length (SL), seedling vigor (SV), and whole seedling length (WSL). These changes resulted in increased carotenoid, 2,2‐Diphenyl‐1‐picrylhydrazyl (DPPH) activity, hydrogen peroxide (H_2_O_2_), and malondialdehyde (MDA) contents in the roots and shoots of the inoculated seedlings. Principal component analysis (PCA) revealed a clear separation between the control and the inoculated seedlings, with the biochemical traits showing a strong association with isolate Fv‐B12024. Additionally, the shoot traits were less responsive to the fungus effects and exhibited low discriminatory power compared to the root biochemical traits. Overall, these findings demonstrated that *F. verticillioides* infection shifted Bihilifa maize seedlings from a high‐vigor physiological state toward a stress‐dominated biochemical state.

## 1 Introduction

*Fusarium verticillioides* (Sacc.) Nirenberg (syn. *F. moniliforme* Sheldon, teleomorph *G. fujikuroi* (Sawada) Wr.) is a primary pathogen of the cereal species maize ( *Zea mays* L.) and is highly toxic and adversely affects seedling performance and human health (Hudu et al. 2024; Mahunu et al. 2024; Ren et al. 2025). The fungus can systematically colonize maize root, stalk, and ear at all developmental stages, and it is the most prolific producer of fumonisin in grains (Murillo‐Williams and Munkvold 2008; Waalwijk et al. 2008; Yli‐Mattila and Sundheim 2022). *Fusarium verticillioides* is a symptomless endophyte that causes diseases in plants' vegetative and reproductive tissues (Rodriguez Estrada et al. 2012). *Fusarium verticillioides* has been isolated and characterized from various tissues of maize and more recently found in tassels (Arata et al. 2024).

In response to *F. verticillioides* infection, host plants produce a wide array of bioactive metabolites including reactive oxygen species (ROS), plant hormones, and photosynthetic pigments (López‐Coria et al. 2023; Xu et al. 2023). For instance, *F. verticillioides* inoculated maize plants showed increased activities of chitinase, β‐1‐3‐glucanase, phenylalanine ammonia‐lyase, polyphenol oxidase, catalase, ascorbate peroxidase, and peroxidase (Cacique et al. 2020). The contents of photosynthetic pigments, including chlorophyll a, chlorophyll b, and carotenoids, decreased in maize seedlings infected with *F. verticillioides*, while stomatal conductance was unaffected (Baghbani et al. 2019). Thus, understanding the physiological and biochemical changes occurring in plants infected by *F. verticillioides* can help manage crop yield losses.

Seed germination is one of the most important processes of a plant's life cycle. However, there has been a wide variance in the apparent effect of *F. verticillioides* infection on germination‐linked traits (Ferrigo et al. 2023; Stagnati et al. 2020). For example, in maize seeds treated with *F. verticillioides*, the germination percent (GP), whole seedling length (WSL), and seed vigor (SV) increased, while the root length (RL) declined, with no variation observed in shoot length (SL) (Harish et al. 2023). Similarly, *F. verticillioides* was reported to impair the emergence rate, vigor, plant population, and length and weight of maize seedlings (Machado et al. 2013). In contrast to these reports, Stagnati et al. (2020) observed an increase in the germination rate of 59 maize genotypes treated with *F. verticillioides*, while 87 had a reduced germination rate.

The wide occurrence of *F. verticillioides* makes evaluating various *F. verticillioides* aggressiveness on maize plants imperative. *Fusarium* species in Africa are widespread, and *F. verticillioides* is the most prevalent species commonly isolated in Ghana (Hudu et al. 2024; Kpodo et al. 2000; Opoku et al. 2024). However, to our knowledge, no study has evaluated the influence and aggressiveness of *F. verticillioides* in maize genotypes commonly grown in Ghana. Therefore, the objectives of this study were to: (i) identify naturally occurring *F*. *verticillioides* isolates from Bihilifa maize seeds in Ghana and (ii) evaluate their effects on seed germination and biochemical responses of maize seedlings.

## 2 Materials and Methods

### 2.1 Plant Materials and Morphological Identification

Seeds of the maize variety Bihilifa (National registration code: GH/Zm/020/15) were obtained from the Savannah Agricultural Research Institute (SARI) in Nyankpala, Ghana. The variety is leaf blight‐tolerant among other genotypes previously reported (Ministry of Food and Agriculture 2019). The *F. verticillioides* isolates were isolated from the seeds using the procedure described by Opoku et al. (2024). Single isolation and morphological characterization were carried out using the methods of Opoku et al. (2011) and Leslie and Summerell (2006), respectively.

### 2.2 Molecular Characterization

Fungi gDNA was extracted using the CTAB (Lee et al. 1988). The extracted gDNA was amplified using primer sequences of the translation elongation factor 1‐alpha gene: forward primer: ATGGGTAAGGAAGACAAGAC and reverse primer: GGAAGTACCAGTGATCATGTT (O’Donnell et al. 1998). The VWR Red Taq DNA Polymerase Master Mix (1.5 mM MgCl2) was used in the polymerase chain reaction (PCR). Polymerase chain reaction (PCR) was carried out using the thermal cycling conditions of Opoku et al. (2024). The PCR products were visualized on a 2% agarose gel stained with 250 μL of 60 × ethidium bromide. The PCR products were sequenced at Inqaba Biotec.

### 2.3 Phylogenetic Analysis

Sequences were edited and cleaned with BioEdit 7.0.5 (Hall 2005). The cleaned sequences were compared against the Fusarioid‐ID database (www.Fusarium.org) and non‐redundant GenBank databases (http://blast.ncbi.nlm.nih.gov/Blast.cgi). Then the obtained sequences were combined with other species in the *Fusarium fujikuroi* species complex (FFSC) for phylogenetic analysis. *F*. *incarnatum* and *F. oxysporum* were used as outgroups in the analysis. Nucleotide sequence alignment was done in Clustal Omega (Madeira et al. 2022). The inferred phylogenetic trees were constructed based on maximum likelihood (ML) and Bayesian inference (BI) methods (Kumar et al. 2016; Ronquist et al. 2012). The ML phylogenetic tree was constructed with MEGA v.12, implementing the Tamura‐Nei model with 1000 bootstrap replicates. The BI tree was constructed in MrBayes v.3.2.7a with the Markov chain Monte Carlo (MCMC) algorithm. The MCMC chains sampled every 100 generations and terminated when the standard deviation of split frequency was less than 0.01 (1,000,000 run). The BI phylogenetic tree was visualized and edited in FigTree v.1.4.4.

### 2.4 Pathogenicity Assay

#### 2.4.1 Seed Sterilization

Seeds of maize variety Bihilifa were used in the pathogenicity assay. Uniform‐sized seeds with no visible damage were surface disinfected in commercial bleach containing 3.5% sodium hypochlorite (Trade name: Madar zone) for 3 min and rinsed three times with sterilized distilled water. To achieve internal sterilization, the seeds were treated with a heat shock at 60°C for 5 min (Glenn et al. 2008).

#### 2.4.2 Seed Inoculation and Germination Assay

Single cultured isolates were collected and diluted to 1 × 10^6^ conidia/mL (López‐Coria et al. 2023). The artificial kernel inoculation method was used to inoculate the sterile maize seeds in a completely randomized design (CRD) with four replications per isolate. One hundred and twenty sterile seeds were randomly selected and immersed in 100 mL of the different *F. verticillioides* suspensions for approximately 10 h at 28°C in the dark (Glenn et al. 2008; Koch et al. 2020). Control seeds were treated with sterile water.

Germination assay was conducted using the Rolled Towel Assay (RTA) (Septiani et al. 2019). Two towels of germination paper were moistened with sterile distilled water. For each replicate, 30 seeds were arranged on one of the paper towels. The second paper towel was carefully placed onto the first paper towel containing the seeds, leaving the seeds sandwiched between the two towels. The paper towels were rolled up and incubated vertically at 27°C for 7 days.

### 2.5 Data Measurements

#### 2.5.1 Germination Traits

To investigate the role of *F. verticillioides* in maize germination traits, germination (GP), seedling vigor (SV), root length (RL), shoot length (SL), and whole seedling length (WSL) were measured. Germination was recorded after 7 days of incubation. Seeds with shoots longer than 2 cm and with at least one strong root were categorized as germinated.

$$\mathrm{GP}=\left(\text{Number of germinated seeds}/\text{Total number of seeds}\right)\times 100$$

SV=GP×WSL

RL, SL, and WSL were measured for 10 randomly selected seedlings per treatment using a digital vernier caliper. The number of roots per treatment was obtained by counting.

#### 2.5.2 Seedling Biomass and Biomass Responses to *Fusarium verticillioides* (BRFv)

The fresh root and shoot biomass per treatment was measured after 7 days of germination. The root and shoot samples were dried at 60°C for 48 h in separate envelopes. The BRFv was calculated using the average seedling weight of each treatment (Wilkins 1978).

$$\text{BRFv}=\frac{\text{Seedling treated with Fusarium verticillioides}}{\text{Seeding treated with water}\;\left(\text{control}\right)}\times 100$$

### 2.6 Biochemical Test

The carotenoid, 2,2‐diphenyl‐1‐picrylhydrazyl (DPPH), hydrogen peroxide (H_2_O_2_), and malondialdehyde (MDA) contents in the roots and shoots were measured from 7‐day‐old germinated maize seedlings. The roots and shoots from each treatment were detached from the seedlings and dried at 60°C for 12 h. The dried tissues were then ground into a fine powder using a laboratory mortar and pestle, and the resulting powder was then used for the biochemical assay.

#### 2.6.1 Determination of Carotenoid Content

The carotenoid content was determined using the procedure described by Md Saleh et al. (2020), with slight modifications, by extracting 0.5 g of powdered dried tissue in a 2.5 mL mixture of hexane, acetone, and ethanol in a 2:1:1 ratio. The mixture was stored for 60 min at 4°C until the sample turned completely white; then it was centrifuged at 3000 rpm for 10 min; the supernatant was transferred into a different tube, and 5 mL of distilled water was added to separate the hexane layer. The upper hexane layer was transferred into new tubes and topped with 10 mL of hexane. The absorbances were determined at 450 nm using a Biobase UV/Vis spectrophotometer (BK‐V1000).

$$\text{Total Carotenoids}\;\left(\mathrm{\mu g}/g\right)\;\text{was expressed}\;\mathrm{as}=\frac{A_{450}\times V\;\left(\mathrm{ml}\right)\times 10^{4}}{A_{1\mathrm{cm}}^{1\%}\times \text{sample weight}\;\left(g\right)}$$

where A_450_ is absorbance; *V* is the total extract volume; and $A_{1\mathrm{cm}}^{1\%}$ = 2500 is the extinction coefficient of carotenoids in hexane.

#### 2.6.2 Determination of 2,2‐Diphenyl‐1‐Picrylhydrazyl (DPPH)

The DPPH scavenging activity in the roots and shoots was determined using the methods of Afsar et al. (2012) with slight modification. Dry root and shoot tissues (0.5 g each) were ground separately in an ice bath with 10 mL of methanol, stored for 24 h in the dark, and the mixture strained using Whatman No. 1 filter paper. Then the obtained extracts were stored for 3 days, and 100 μL of each extract was combined with 900 μL of methanol and 4 mL of DPPH. The mixtures were incubated for 30 min at room temperature in the dark, and the absorbance was measured at 517 nm using a UV–visible spectrophotometer (BK‐V1000 Spectrophotometer). DPPH and methanol were used as the blank control, and ascorbic acid was used as standard. The scavenging activity was calculated as [(AC−AS)/AC] × 100. Where, AC = Absorbance of the control solution, AS = Absorbance of the maize extracts.

#### 2.6.3 Determination of Hydrogen Peroxide (H_2_O_2_ ) Content

The H_2_O_2_ and content in the germinated roots and shoots were measured using the method of Shahzad et al. (2021) with slight modifications to the extract method. Briefly, 250 μL of methanolic extracts (1.25 mg/μL) was added to 250 μL of 0.1% Trichloroacetic acid (TCA), 1 mL of 1 M potassium iodide, and 0.5 mL of potassium phosphate buffer (pH 7.4). The mixture was placed under constant temperature (28°C) for 1 h. The absorbance was measured at 390 nm.

#### 2.6.4 Determination of Malondialdehyde (MDA) Content

Contents of MDA in root and shoot samples were measured using the procedure described by Hodges et al. (1999). One milliliter of methanolic extracts (5 mg/μL) was added to a test tube containing 1 mL of either (i) –thiobarbituric acid (TBA) solution comprised of 20.0% (w/v) trichloroacetic acid (TCA) and 0.01% butylated hydroxytoluene (BHT), or (ii) +TBA solution containing the above plus 0.65% TBA. The mixtures were vortexed vigorously and heated for 25 min at 95°C. The solutions were cooled and centrifuged for 10 min at 3000 rpm, and the absorbance was read at 440, 532 and 600 nm. Finally, the MDA contents were calculated using the formula of Hodges et al. (1999).

### 2.7 Statistical Analysis

Data for the germination‐linked traits and biochemical compounds were subjected to one‐way analysis of variance (ANOVA) using GenStat statistical software (12th edition). The Duncan multiple range test was used to compare means when the ANOVA was significant at 0.05. Principal component analysis (PCA) and Pearson correlation test were carried out using XLSTAT software (2021.2.2). Correlation analysis was performed between germination‐linked traits and biochemical compounds.

## 3 Results

### 3.1 Morphological and Phylogenetic Analysis

A total of six fungal cultures, including three *Fusarium* isolates (Fv‐B12024, Fv‐B22024, and Fv‐B32024), were obtained from the maize samples (Bihilifa). Based on the observation of the colony morphology on PDA and conidia characteristics (results not shown), the *Fusarium* isolates were preliminarily identified as *F. verticillioides*.

**FIGURE 1:**
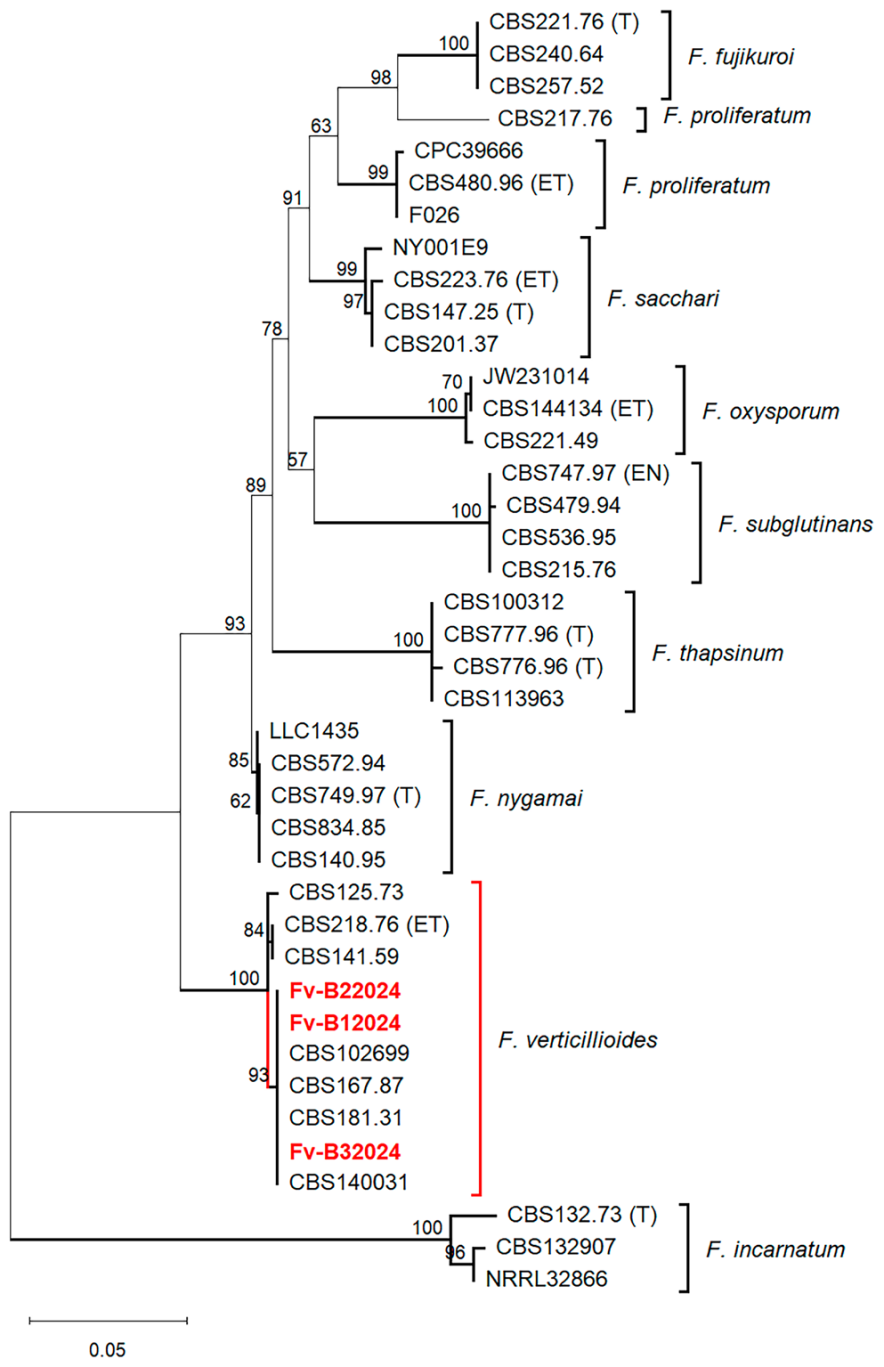
Maximum likelihood phylogenetic tree based on the *translation elongation factor 1 alpha* (*TEF1‐α*) gene sequences showing the relationship among *Fusarium fujikuroi* species complex. Ex‐type (T), ex‐epitype (ET) and ex‐neotype strains (EN) (retrieved from the CBS collection) are indicated by their culture codes. Numbers at nodes represent bootstrap support values (1000 replicates). Strains marked with Fv‐B12024, Fv‐B22024, and Fv‐B32024 represent isolates obtained in the present research.

To further confirm the identity of the *F. verticillioides* isolates, the TEF1‐α gene sequences of the three isolates were initially aligned against the Fusarioid‐ID database (www.Fusarium.org) and non‐redundant GenBank databases (http://blast.ncbi.nlm.nih.gov/Blast.cgi). The closest match showed a 99.50% sequence similarity to *F. verticillioides* CBS 218.76, the designated ex‐epitype strain of *F. verticillioides*. Both the ML (Figure 1) and BI (Figure S1) phylogenetic analyses yielded highly similar (congruent) topologies. Therefore, ML phylogeny was adopted as the basal tree for illustrating and comparing the phylogenetic positioning of the three *F. verticillioides* isolates. Similar to the BLAST results, the ML phylogenetic analysis of the TEF1‐α gene clustered all three isolates within the *F. verticillioides* ex‐epitype clade with strong bootstrap and posterior probabilities support (BS/PP = 100%/1.0). This finding further confirms the identity of the three isolates as *F. verticillioides*. All obtained sequences of the present *F. verticillioides* isolates were deposited in GenBank (https://www.ncbi.nlm.nih.gov/) under the accession numbers presented in Table S1.

The branching order between *F. fujikuroi* and *F. proliferatum* was only partially resolved (Figure 1), as indicated by a relatively low bootstrap value of 63%. Consequently, *F. proliferatum* strain CBS 217.76 formed a strongly supported sister relationship with *F. fujikuroi* (98% bootstrap). The *F. oxysporum* outgroup was connected to the tree close to *F. subglutinans*; however, the branching order between these species remained unresolved (57% bootstrap). In contrast, the *F. incarnatum* outgroup root provided a completely resolved phylogeny within the *F. fujikuroi* species complex.

### 3.2 Effect of *F. verticillioides* on Germination‐Linked Traits

**FIGURE 2:**
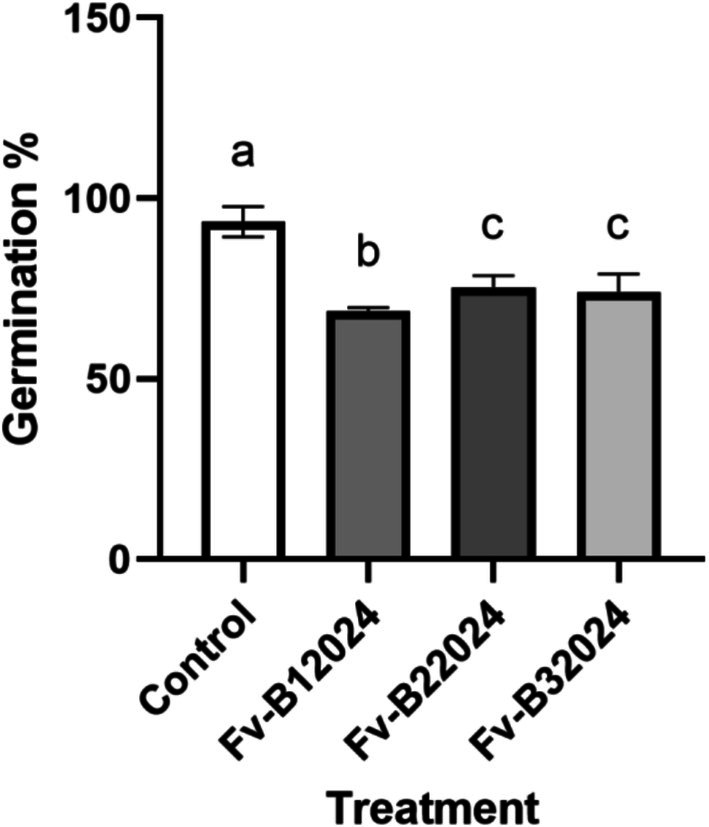
Effect of *Fusarium verticillioides* on germination percent. Bar value represents mean ± SD of three replications. Bar values with the different letters significantly differ at *p* < 0.05.

**FIGURE 3:**
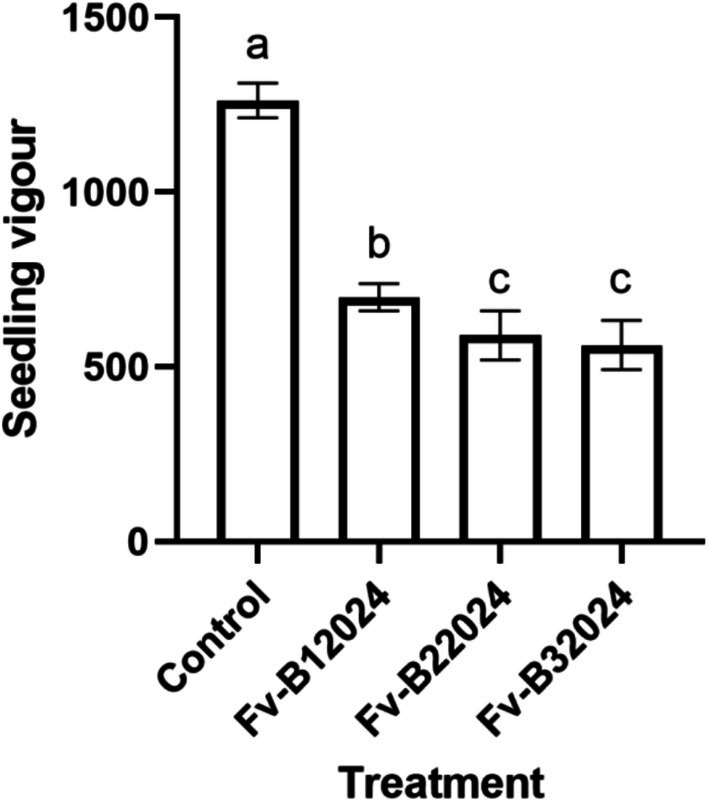
Effect of *Fusarium verticillioides* on seedling vigor. Bar value represents mean ± SD of three replications. Bar values with the different letters significantly differ at *p* < 0.05.

The effect of *F. verticillioides* on germination‐linked traits was assessed by measuring the GP, SV, RL, SL, and WSL. Relative to the control treatment, seeds inoculated with the *Fusarium* isolates had lower GP, poor SV, shorter RL, SL, and WSL, and a lower number of roots. Although isolate Fv‐B12024 had the lowest GP among the *F. verticillioides* treated seeds (Figure 2), Fv‐B12024 had strong seedling vigor, primarily through increasing the WSL (Figure 3). The GP and SV in the Fv‐B22024 and Fv‐B32024 treated seedlings were similar (*p* > 0.05).

**FIGURE 4:**
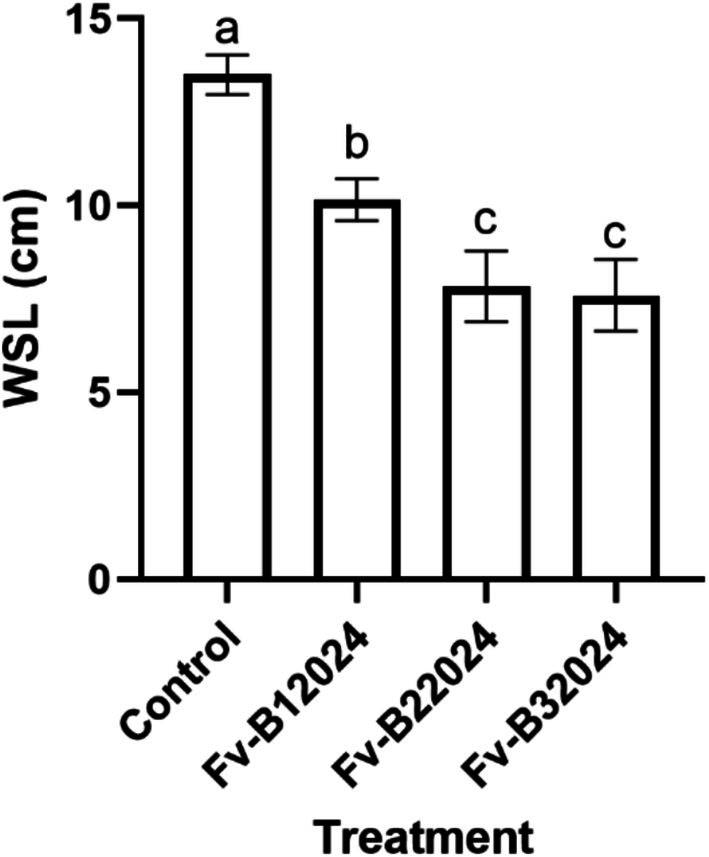
Effect of *Fusarium verticillioides* on whole seedling length (WSL) on maize seedlings. Bar value represents mean ± SD of three replications. Bar values with the different letters significantly differ at *p* < 0.05.

**FIGURE 5:**
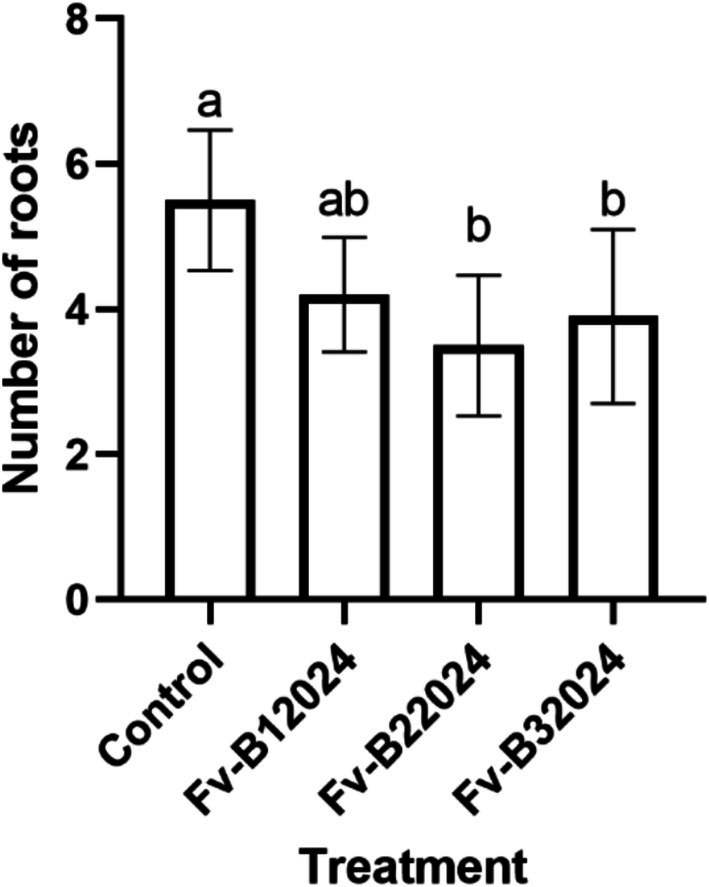
Effect of *Fusarium verticillioides* on the number of roots on maize seedlings. Bar value represents mean ± SD of three replications. Bar values with the different letters significantly differ at *p* < 0.05.

The number of roots and WSL were significantly (*p* = 0.0005) affected by the treatments (Figures 4 and 5). Seeds treated with Fv‐B12024, Fv‐B22024, and Fv‐B32024 had reduced WSL, primarily by reducing the length of the primary root and shoot. Relative to the control seedlings, isolate Fv‐B12024 decreased the WSL and number of roots by 24.74% and 23.64%, respectively. However, the decrease in the number of roots in Fv‐B12024 treated seedlings was comparable (*p* > 0.05) to control seedlings. While Fv‐B12024 treated seedlings had significantly longer whole seedling lengths than Fv‐B22024 and Fv‐B32024, there were no significant differences among the *F. verticillioides*‐treated seedlings for the number of roots.

**FIGURE 6:**
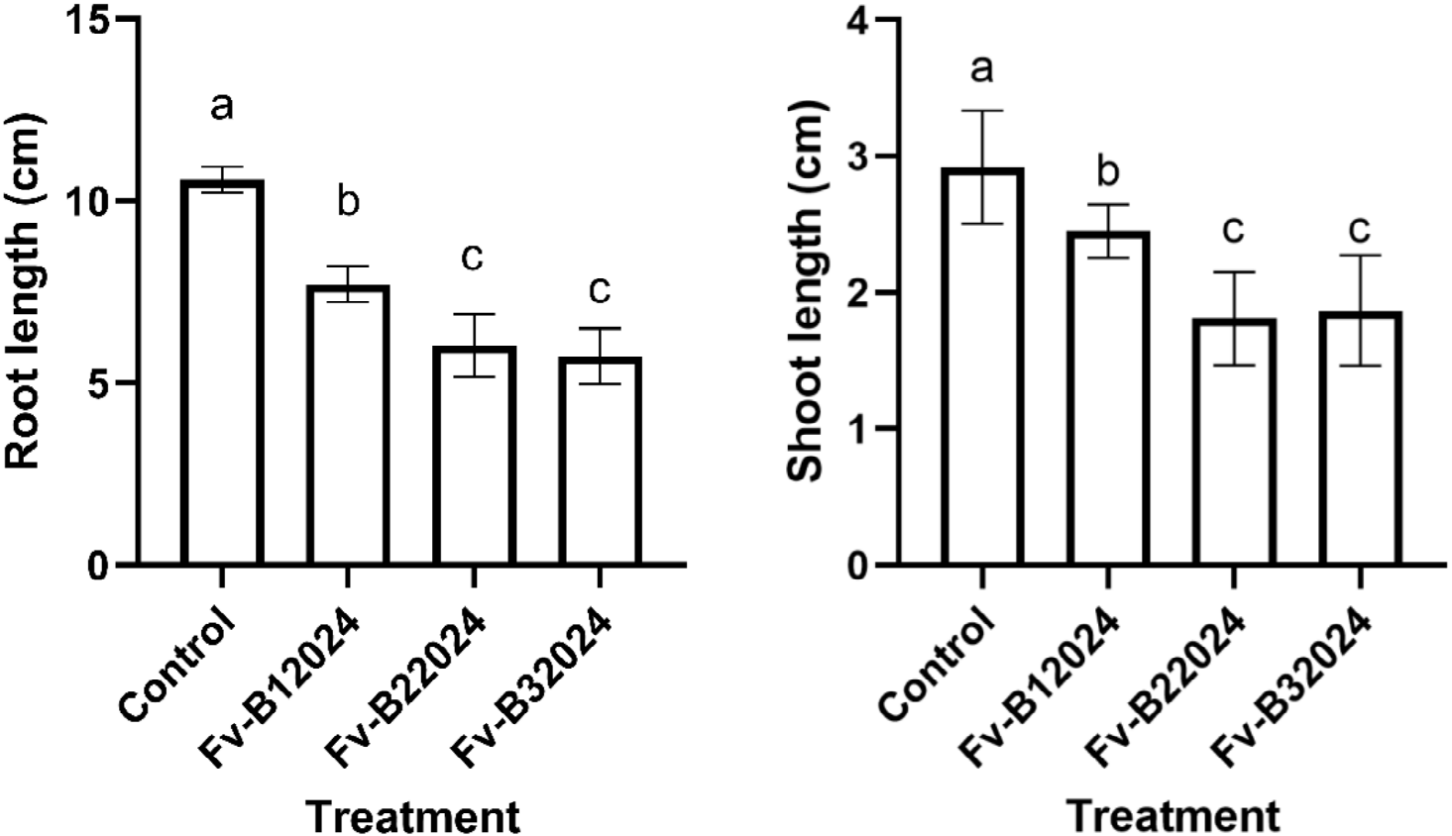
Effect of *Fusarium verticillioides* on root and shoot lengths of maize seedlings. Bar value represents mean ± SD of three replications. Bars values with the different letters significantly differ at *p* < 0.05.

*Fusarium verticillioides* inoculation significantly (*p* < 0.0001) reduced the root and shoot elongation of maize seedlings (Figure 6). Relative to the control seedling, isolates Fv‐B12024, Fv‐B22024, and Fv‐B32024 decreased the RL by 27.13%, 43.01%, and 45.84%, respectively. Similarly, *F. verticillioides* significantly reduced the shoot SL of the seedlings. Isolate Fv‐B22024 and Fv‐B32024 treated seedlings had the least SL (*p* > 0.05) of 38.01% and 35.96%, respectively.

### 3.3 Effect of *Fusarium verticillioides* Isolates on Biochemical Characteristic of Maize

#### 3.3.1 DPPH Content

**FIGURE 7:**
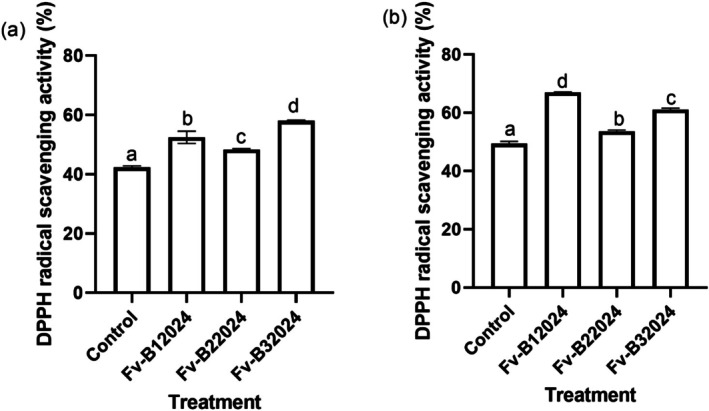
Effect of *Fusarium verticillioides* on root (a) and shoot (b) DPPH radical scavenging activity of maize seedlings. Bar values represent mean ± SD of three replications. Bars values with the different letters significantly differ at *p* < 0.05.

The contents of DPPH radical scavenging activity were significantly (*p* < 0:001) affected by the treatments. Higher DPPH activities were observed in the shoots than in the roots. Relative to the control plants, Fv‐B12024, Fv‐B22024, and Fv‐B32024 inoculation increased the content of DPPH scavenging activity in the seedling roots by 19.27%, 12.52%, and 27.08%, respectively (Figure 7).

#### 3.3.2 Carotenoid Content

**FIGURE 8:**
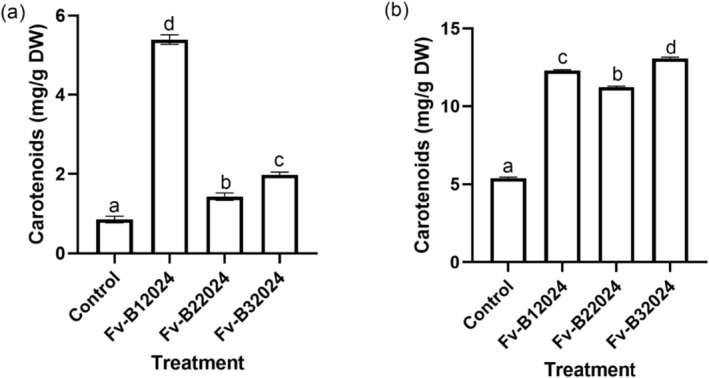
Effect of *Fusarium verticillioides* on root (a) and shoot carotenoid (b) content of maize seedlings. Bar values represent mean ± SD of three replications. Bars values with the different letters significantly differ at *p* < 0.05.

*Fusarium verticillioides* isolates significantly increased the carotenoid content in germinated seeds, roots, and shoots, with high carotenoids content observed in the shoots. In comparison with the control seedlings, the seedling carotenoid concentration in the roots and shoots was respectively increased by 84.13% and 56.24% for Fv‐B12024, 40.11% and 52.14% for Fv‐B22024, and 56.69% and 58.87% for Fv‐B32024 (Figure 8). The greatest increase in carotenoid content was observed in the Fv‐B12024 isolate for roots, while the Fv‐B32024 isolate had the highest carotenoid content in the shoots.

#### 3.3.3 Hydrogen Peroxide (H_2_O_2_ ) and Malondialdehyde (MDA) Contents

**FIGURE 9:**
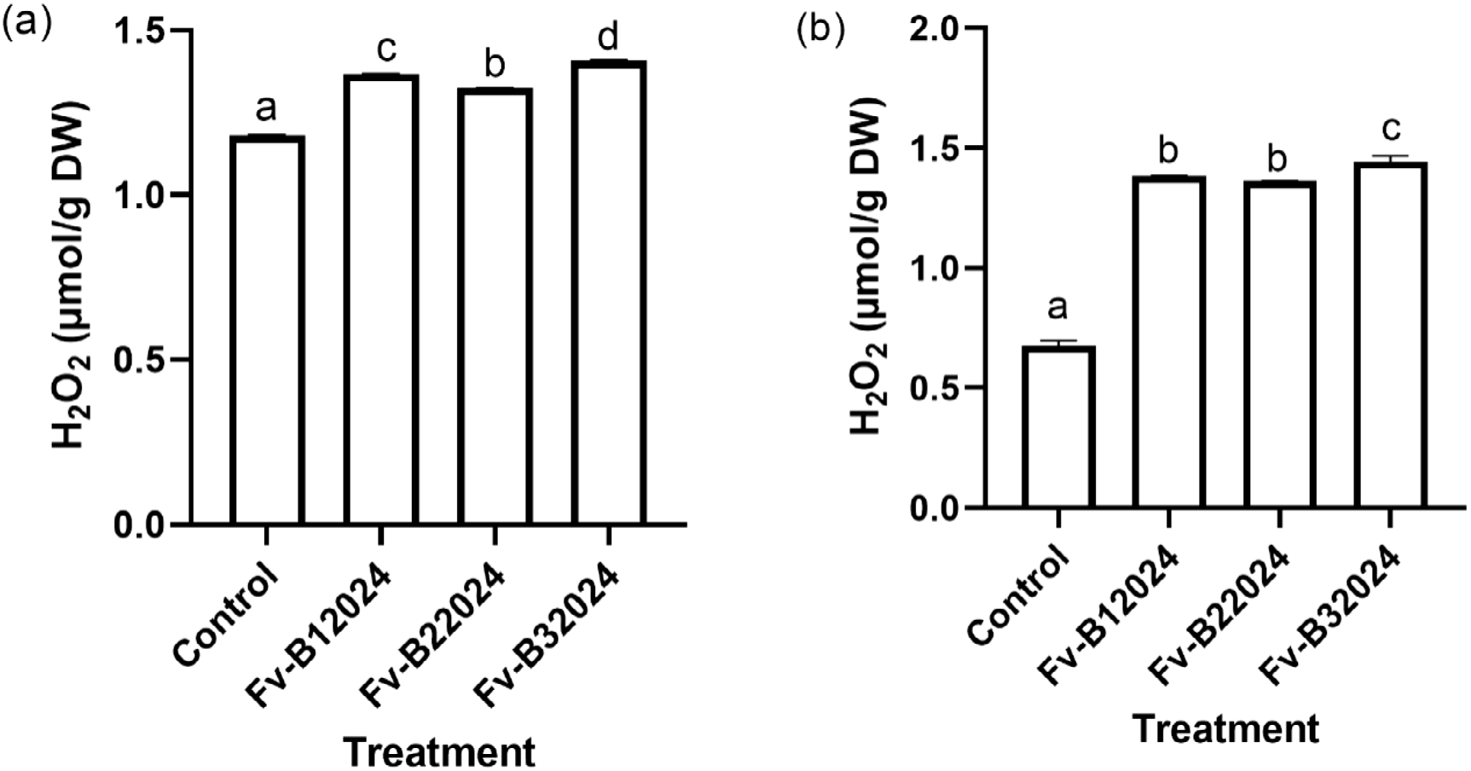
Effect of *Fusarium verticillioides* on root (a) and shoot (b) H_2_O_2_ content of germinated seeds. Bar values represent mean ± SD of three replications. Bars values with the different letters significantly differ at *p* < 0.05. WSL.

The effect of *F. verticillioides* on oxidative stress was determined by measuring the contents of H_2_O_2_ and MDA in the root and shoot of the germinated seeds. Relative to control seedlings, all the isolates significantly increased H_2_O_2_ content. Isolate Fv‐B32024 had the highest H_2_O_2_ content in both the roots and shoot (Figures 9a,b). Except for the control treatments, the H_2_O_2_ content was higher in the shoot compared to the root.

**FIGURE 10:**
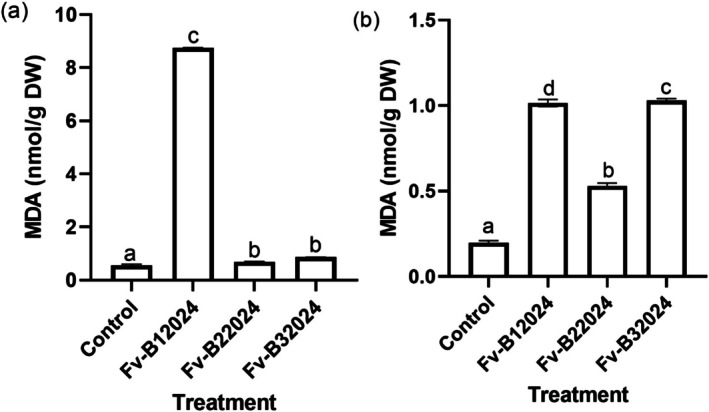
Effect of *Fusarium verticillioides* on root (a) and shoot (b) MDA content of germinated maize seeds. Bar values represent mean ± SD of three replications. Bars values with the same letters are significantly different at *p* < 0.05.

All the *F. verticillioides* treated seedlings accumulated more MDA than the control (Figures 10a,b). Isolate Fv‐B12024 treated seedlings had the highest MDA content in the root and shoot.

### 3.4 Biomass and Biomass Response to *Fusarium verticillioides* (BRFv) of Maize Seedling

Relative to the control seedlings, all the *F. verticillioides* treated seedlings had significantly lower fresh root weight (39.44%–0.24%), dry root weight (31.22%–45.15%), and dry shoot weight (22.44%–28.95%). However, the difference between *F. verticillioides* treated seedlings and the control seedling was not significant for fresh shoot weight (Table 1). In addition, the biomass response to *F. verticillioides* (BRFv) of the Fv‐B22924 treated seedling was the highest (77.30%). The seedling fresh and dry shoot weight suffered a lesser reduction to BRFv when compared with the root system, indicating that the root suffered greater stress from the *F. verticillioides* treatment.

**TABLE 1:** Effect of treatments on root and shoost biomass and plant biomass response to *Fusarium. verticillioides* (BRFv) at 7 days after germination.

Treatment	Fresh weight (g)		Dry weight (g)		
	Root	Shoot	Root	Shoot	BRFv (%)*
Control	16.30 ± 2.66^a^	20.42 ± 2.95	2.37 ± 0.17^a^	2.51 ± 0.29^a^	100
Fv‐B12024	8.11 ± 0.11^b^	16.34 ± 0.13	1.30 ± 0.16^b^	1.78 ± 0.32^b^	66.19
Fv‐B22024	9.62 ± 0.36^b^	19.03 ± 1.77	1.57 ± 0.18^b^	1.94 ± 0.05^c^	77.30
Fv‐B32024	9.87 ± 0.22^b^	16.29 ± 0.93	1.63 ± 0.18^b^	1.95 ± 0.15^d^	71.47
*p*‐value	< 0.001	0.053	< 0.001	0.022	

*Note:* Values are treatment means ± SD of three replications. Values within a column with the same superscript letters indicate no significant differences at *p* < 0.05.

* BRFv was not subjected to analysis of variance.

### 3.5 Principal Component Analysis and Pearson's Correlation Analysis

Principal component analysis (PCA) partitioned the traits into five main principal components (PCs) with the first three components having eigenvalues above one. These five PCs accounted for a total of 97.812% of the total variability existing among the traits. Eigenvalues ranged from 11.254 for PC1 through to 0.349 for PC5 (Table 2). While PC1 contributed more than half (62.525%) of the total variation observed, PC2 accounted for 20.966%. PC3, PC4, and PC5 also explained 9.06%, 3.274%, and 1.941% of the variation, respectively (Table 2).

**TABLE 2:** Principal component analysis (PCA) showing the contributions of each trait to the variation.

Traits	PC1	PC2	PC3	PC4	PC5
Germination %	−0.975	−0.036	0.169	0.058	0.008
Root length	−0.820	0.555	−0.017	−0.077	0.002
Shoot length	−0.677	0.717	0.042	−0.001	−0.075
Whole seedling length	−0.801	0.592	−0.003	−0.064	−0.014
Seedling vigor	−0.921	0.374	0.063	−0.068	−0.004
Number of roots	−0.661	0.390	0.340	0.451	−0.070
Root DPPH (%)	0.869	−0.119	0.456	−0.106	−0.036
Shoot DPPH (%)	0.868	0.470	0.121	−0.100	−0.023
Root carotenoid	0.665	0.727	−0.169	−0.027	0.013
Shoot carotenoid	0.983	−0.149	0.058	0.020	−0.054
Root H_2_O_2_	0.516	0.818	−0.246	−0.012	0.036
Shoot H_2_O_2_	0.932	0.184	0.290	−0.074	−0.078
Root MDA	0.501	0.824	−0.257	−0.011	0.038
Shoot MDA	0.335	0.315	0.851	−0.214	−0.103
Root fresh weight	−0.913	0.011	0.245	0.145	0.025
Shoot fresh Weight	−0.723	−0.189	−0.394	−0.347	−0.376
Root dry weight	−0.915	−0.075	0.247	−0.093	−0.138
Shoot dry weight	−0.798	−0.041	0.152	−0.379	0.391
Eigenvalue	**11.254**	**3.774**	**1.639**	**0.589**	**0.349**
Variability (%)	**62.525**	**20.966**	**9.106**	**3.274**	**1.941**
Cumulative %	**62.525**	**83.491**	**92.597**	**95.871**	**97.812**

Abbreviations: H_2_O_2_, Hydrogen peroxide, MDA; Malondialdehyde.

All the germination and biomass‐linked traits contributed negatively to the variation observed in PC1. Traits such as shoot carotenoid, root and shoot DPPH, and root and shoot H_2_O_2_ were the main contributors to the variation observed in PC1; root MDA, root carotenoids, and shoot MDA were identified as the major contributors to PC2 and PC3, respectively.

**FIGURE 11:**
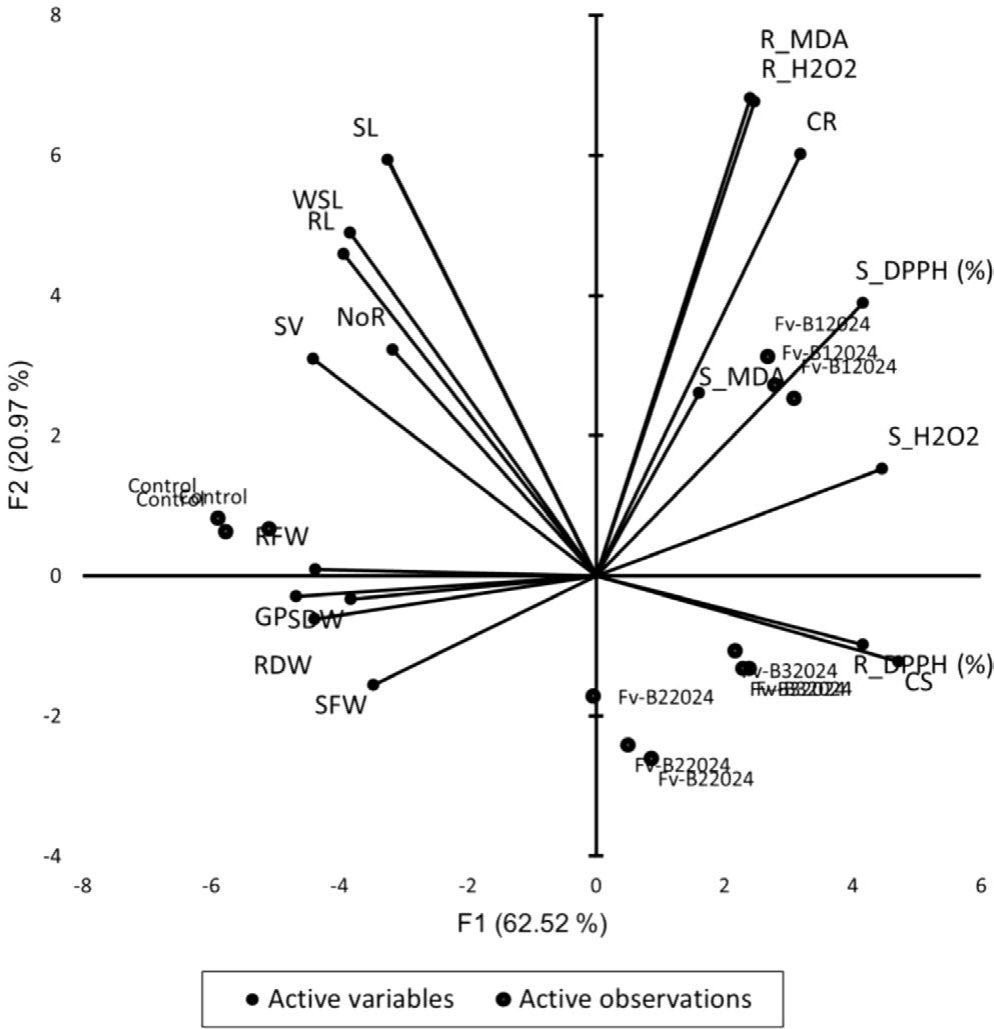
Biplot analysis of PC1 and PC2 showing germination‐linked traits and biochemical compounds. CR, root carotenoid content; CS, shoot carotenoid content; GP, germination %; NoR, number of roots; R_DPPH (%), root DPPH content; R_H2O2, root hydrogen peroxide; R_MDA, root malondialdehyde content; RL, root length; S_DPPH (%), shoot DPPH content; S_H2O2, shoot hydrogen peroxide; S_MDA, shoot malondialdehyde content; SL, shoot length; SV, seeding vigorvigour; WSL, whole seedling length.

Visualization of the principal component biplot indicated trait contributions to variation represented by vectors and associations between traits (Figure 11). The root MDA content, root carotenoid content, and shoot DPPH content were the traits that contributed substantially to the variability among the *F*. *verticillioides* isolates. Isolate Fv‐B12024 in the right upper quadrant strongly associated with traits such as root MDA, shoot MDA, root H_2_O_2_, and shoot H_2_O_2_ contents, root carotenoids, and shoot DPPH. Isolates Fv‐B22024 and Fv‐B32024 in the lower right quadrant are associated with shoot carotenoid and root DPPH contents (Figure 11).

There was a strong negative correlation between the GP and root DPPH (*r* = −0.77, *p* = 0.003), shoot DPPH (r = −0.85, *p* = 0.001), root carotenoid (*r = −*0.704, *p =* 0.011), shoot carotenoid (*r =* −0.94, *p =* 0.0001), root H_2_O_2_ (*r =* −0.58, *p =* 0.049) and shoot H_2_O_2_ (*r =* −0.87, *p =* 0.000) (Table 3, Table S2). Similarly, RL, SL, and SV correlated negatively (*p* < 0.05) with R_DPPH, CS, R_H_2_O_2_, and S_H_2_O_2_ (Figure 11, Table S2).

**TABLE 3:** Pearson correlations *p*‐value between germination‐linked traits and biochemical compounds in seedlings root and shoot.

Variables	GP	RL	SL	WSL	SV	NoR	R_DPPH	S_DPPH	CR	CS	R_H_2_O_2_	S_H_2_O_2_	R_MDA	S_MDA
GP	**0**													
RL	**0.003**	**0**												
SL	**0.023**	**< 0.0001**	**0**											
WSL	**0.005**	**< 0.0001**	**< 0.0001**	**0**										
SV	**< 0.0001**	**< 0.0001**	**0.000**	**< 0.0001**	**0**									
NoR	**0.011**	**0.012**	**0.004**	**0.009**	**0.007**	**0**								
R_DPPH	**0.003**	**0.003**	**0.022**	**0.004**	**0.001**	0.088	**0**							
S_DPPH	**0.001**	0.145	0.451	0.184	**0.035**	0.211	0.004	0						
CR	**0.011**	0.672	0.848	0.757	0.266	0.480	0.177	**< 0.0001**	0					
CS	**< 0.0001**	**< 0.0001**	**0.004**	**0.000**	**< 0.0001**	**0.019**	**< 0.0001**	**0.002**	0.073	**0**				
R_H_2_O_2_	**0.049**	0.906	0.490	0.822	0.568	0.709	0.449	0.002	< 0.0001	0.238	**0**			
S_H_2_O_2_	**0.000**	**0.018**	0.116	**0.027**	**0.004**	0.131	**< 0.0001**	**< 0.0001**	**0.010**	**< 0.0001**	0.060	**0**		
R_MDA	0.056	0.869	0.463	0.786	0.600	0.728	0.485	0.002	< 0.0001	0.260	< 0.0001	0.069	**0**	
S_MDA	0.525	0.768	0.900	0.836	0.715	0.779	**0.019**	0.055	0.324	0.296	0.497	0.026	0.523	**0**

*Note:* Values in bold are different from 0 with a significance level of alpha = 0.05.

Abbreviations: CR, root carotenoid content; CS, shoot carotenoid content; GP, germination %; NoR, number of roots; R_DPPH (%), root DPPH content; R_MDA, root malondialdehyde content; RL, root length; S_DPPH (%), shoot DPPH content; S_MDA, shoot malondialdehyde content; SL, shoot length; SV, seeding vigor; WSL, whole seedling length.

## 4 Discussion

The present study extends earlier investigations in Ghana that have characterized *F. verticillioides* as the most prevalent *Fusarium* species in Ghanaian maize varieties (Korley et al. 2022; Kpodo et al. 2000; Opoku et al. 2024). The Bihilifa maize variety is commonly grown in the northern parts of Ghana (Asante, Appiah, et al. 2024; Asante, Obed, et al. 2024). This is the first characterization of host biochemical profile that is responsive to *F*. *verticillioides* infection in Ghanaian maize variety, and it is the first step in exploring host immunity against the pathogen.

The rolled towel assay (RTA) was used to evaluate the effects of the *F. verticillioides* isolates on seed germination and seedling performance. The RTA has been demonstrated to effectively assess maize seedling resistance to *F. verticillioides*, offering reliable visual indicators of infection (Septiani et al. 2019; Tran et al. 2024). Furthermore, studies have shown a strong antagonistic relationship between maize roots and stalks with *F. verticillioides*. Thus, the stalk and root, rather than the ear, serve as the primary sites for antagonism and coevolution between maize and *F. verticillioides* (Xiong et al. 2024).

There have been conflicting reports on the impact of *F. verticillioides* on seedling performance: GP, SV, RL, and SL, primarily due to the varietal tolerance levels and the aggressiveness of different *F. verticillioides* isolates (Harish et al. 2023; Román et al. 2020). In this study, the *F. verticillioides* isolates significantly reduced seed germination, SV, RL, and SL, the number of roots, and biomass accumulation. This observation is consistent with the findings of Stagnati et al. (2020), who reported that *F. verticillioides* inoculation reduced germination by up to 70% in certain maize inbred lines, including I29 (popcorn) and IL677a (Sweet corn), both of which exhibited increased disease severity following infection.

It has been shown that naturally contaminated maize seeds with a high incidence of *F. verticillioides* showed no significant reduction in germination rate (Danielsen and Jensen 1998; Machado et al. 2013). However, several in vitro studies point to the direction that *F. verticillioides* inoculation decreased seed GP, RL, and SL (Harish et al. 2023; Navale et al. 2023; Stagnati et al. 2020). These variations between naturally and in vitro contaminated seeds could partly be explained by concentration levels (Koch et al. 2020). In this study, the 1 × 10^6^ concentration also decreased the GP, RL, and SL of treated seedlings. Although the fumonisin‐producing capacity of the current isolates was not determined, it is worth mentioning that *F. verticillioides* isolates with high fumonisin‐producing ability cause severe reduction in seed germination (Covarelli et al. 2012; Danielsen and Jensen 1998).

*Fusarium verticillioides* induced stress increases lipid peroxidation, characterized by increased accumulation of hydrogen peroxide (H_2_O_2_) and malondialdehyde (MDA), in plant cells (Lanubile et al. 2012; Xu et al. 2022). These increases in the accumulation of H_2_O_2_ and MDA often retard the germination and development of plants. In this study, H_2_O_2_ and MDA levels were negatively correlated with all germination‐linked traits (Figure 7), indicating that increased oxidative damage was associated with reduced seedling vigor. This observation is consistent with the findings of Cacique et al. (2020) who reported that *F. verticillioides* inoculation increased the content of MDA in the internodes and nodes of maize seedlings. The relatively high H_2_O_2_ content in germinated roots and shoots compared to MDA content supports the idea that H_2_O_2_ is the most important ROS involved in lipid peroxidation during *F. verticillioides* infection (Antić et al. 2020). In other experiments, seed treatment with F. verticillioides decreased maize seed germination (Sun et al. 2025) and increased MDA content in the leaves (Yang et al. 2025).

Carotenoids play a critical role in scavenging free radicals, thereby mitigating oxidative stress and preserving cellular integrity under adverse conditions. Chrpová et al. (2021) reported that adequate high‐quality antioxidants are crucial for neutralizing the harmful effect of free radicals generated during a pathogen attack. An increase in the carotenoids and DPPH content was observed, confirming the response to the increased H_2_O_2_ and MDA levels. This increase in carotenoid contents and DPPH scavenging activity, though not sufficient to mitigate the negative impact of *F. verticillioides* on the seedlings’ performance, compares well with the findings that maize plants produced varied antioxidants to defend themselves against *F. verticillioides* attack (Kaur et al. 2022). However, this result is in contrast with the findings of Baghbani et al. (2019), who reported decreased contents of carotenoids in maize seedlings exposed to *F. verticillioides* (2 × 10^5^ per mL). The strong negative correlations observed between the germination‐linked traits and the biochemical compounds in the germinated seed roots and shoots suggest that *F. verticillioides* impairs seed germination by inducing high production of ROS in plants (Lanubile et al. 2012; Li et al. 2022; Thiruvengadam et al. 2024).

According to Nouioura et al. (2024), vectors with smaller angles between them in a PCA biplot indicate strong correlations among variables. In the present study, the results of the H_2_O_2_, DPPH, MDA, and carotenoid assays revealed a strong negative correlation between the biochemical content and GP as well as the biomass response to *F. verticillioides*. These results are consistent with previous reports showing that elevated H_2_O_2_ and MDA levels are associated with reduced seed germination and seedling development (Ma et al. 2024; Szopińska 2014). The length of the vectors in a biplot measures how well a trait contributes to variability such that traits with longer vectors are the best contributors to total variability observed (Yan and Tinker 2005). In the present study, shoot MDA content and number of roots were the lowest contributors to the total variability due to their short vector length.

## 5 Conclusions

The results of this study have shown that *F. verticillioides* infection induced oxidative stress in the maize genotype Bihilifa, as evidenced by increased levels of H_2_O_2_ and MDA. The infection also elevated the antioxidant activity of seedlings, reflected by higher DPPH scavenging activity and carotenoids levels. PCA further showed that isolate Fv‐B12024 clustered closely with traits associated with oxidative damage and antioxidant response, including root and shoot MDA, root and shoot H_2_O_2_, root carotenoids, and shoot DPPH activity.

## Funding

The authors have nothing to report.

## Conflicts of Interest

The authors declare no conflicts of interest.

## Supporting information


**Figure S1:** Bayesian Inference (BI) phylogenetic tree based on TEF1‐α gene sequences of *Fusarium* species. Posterior probabilities (0.5–1.0) are shown at the nodes. The three isolates (Fv‐B12024, Fv‐B22024, and Fv‐B32024) clustered within the *Fusarium verticillioides* ex‐epitype clade, confirming their identity. *Fusarium oxysporum* and *Fusarium incarnatum* were used as outgroup taxa.

**Table S1:** pei370104‐sup‐0002‐Tables.docx. *Fusarium* strains used in this study and their GenBank accession numbers.

## Data Availability

The data that support the findings of this study are openly available in National Center for Biotechnology Information at https://www.ncbi.nlm.nih.gov/, reference number PQ729884‐PQ729886.
