# Supplementary material for: Identification of Fusarium verticillioides Isolates and Their Impact on Seed Germination and Biochemical Profiles in Maize
Source: Plant Environ Interact. 2025 Dec 23;6(6):e70104. doi: 10.1002/pei3.70104 (PMC12724013; doi:10.1002/pei3.70104)
Supplement: Supplementary file 1 — Figure S1: Bayesian Inference (BI) phylogenetic tree based on TEF1‐α gene sequences of Fusarium species. Posterior probabilities (0.5–1.0) are shown at the nodes. The three isolates (Fv‐B12024, Fv‐B22024, and Fv‐B32024) clustered within the Fusarium verticillioides ex‐epitype clade, confirming their identity. Fusarium oxysporum and Fusarium incarnatum were used as outgroup taxa. [file PEI3-6-e70104-s001.docx]

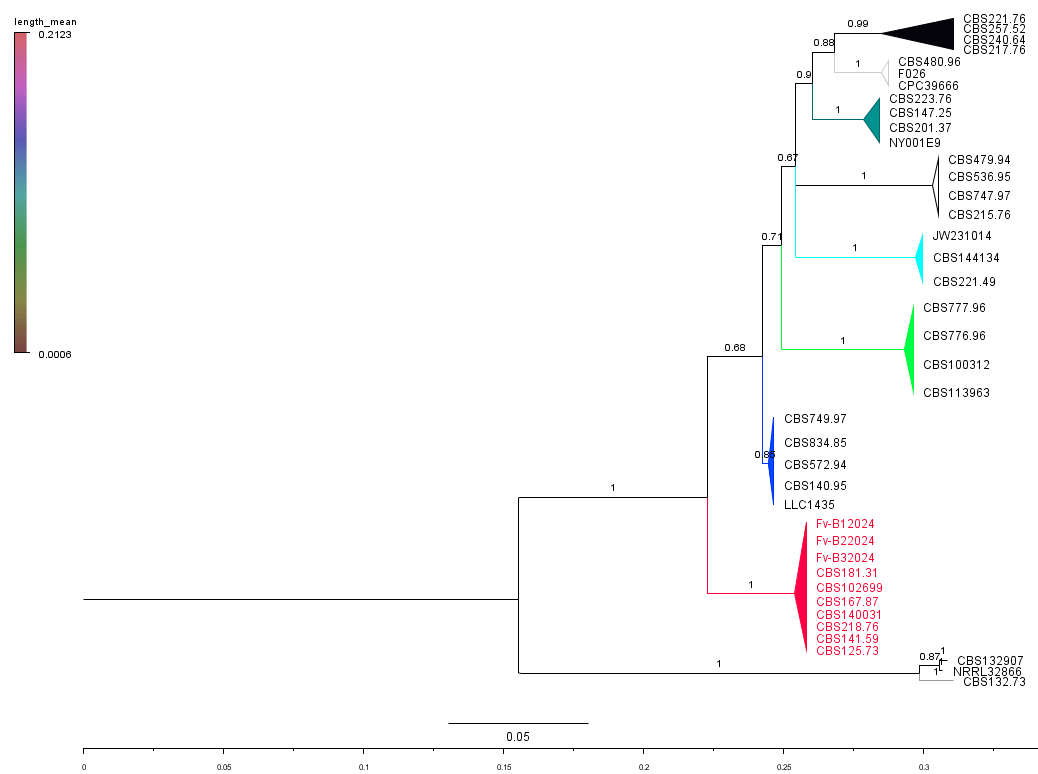


**Supplementary Figure 1:** Bayesian Inference (BI) phylogenetic tree based on TEF1-α gene sequences of Fusarium species. Posterior probabilities (0.5–1.0) are shown at the nodes. The three isolates (Fv-B12024, Fv-B22024, and Fv-B32024) clustered within the Fusarium verticillioides ex-epitype clade, confirming their identity. F. oxysporum and F. incarnatum were used as outgroup taxa
