# Supplementary material for: Identification of Fusarium verticillioides Isolates and Their Impact on Seed Germination and Biochemical Profiles in Maize
Source: Plant Environ Interact. 2025 Dec 23;6(6):e70104. doi: 10.1002/pei3.70104 (PMC12724013; doi:10.1002/pei3.70104)
Supplement: Supplementary file 2 — Table S1: pei370104‐sup‐0002‐Tables.docx. Fusarium strains used in this study and their GenBank accession numbers. [file PEI3-6-e70104-s002.docx]

**Supplementary Table 1:** *Fusarium* strains used in this study and their GenBank accession numbers.

| **Species** | **Ex-/Type strain (culture)** | **GenBank accession number (*TEF1-α*)** | **Note / source** |
| --- | --- | --- | --- |
| *Fusarium verticillioides* | CBS 218.76 | KF499582 | Ex‐epitype |
|  | CBS 102699 | MW401964 |  |
|  | CBS 141.59 | MW402080 |  |
|  | CBS 167.87 | MW402101 |  |
|  | CBS 125.73 | MW402012 |  |
|  | CBS 140031 | MW402076 |  |
|  | CBS 181.31 | MW402103 |  |
|  | **Fv-B12024** | **PQ729884** |  |
|  | **Fv-B22024** | **PQ729885** |  |
|  | **Fv-B32024** | **PQ729886** |  |
| *Fusarium nygamai* | CBS 749.97 | MT011009 | Ex-type |
|  | CBS 834.85 | MW402154 |  |
|  | CBS 572.94 | MW402141 |  |
|  | CBS 140.95 | MW402075 |  |
|  | LLC1435 | OP487015 |  |
| *F. thapsinum* | CBS 776.96 | MN534044 | Ex-type |
|  | CBS 100312 | MW401961 |  |
|  | CBS 777.96 | MW928844 | Ex-type |
|  | CBS 113963 | MW401970 |  |
| *Fusarium subglutinans* | CBS 747.97 | HM057336 | Ex-neotype |
|  | CBS 215.76 | MN534061 |  |
|  | CBS 479.94 | MN534036 |  |
|  | CBS 536.95 | MW402139 |  |
| *F. sacchari* | CBS 223.76 | AF160278 | Ex-epitype |
|  | CBS 147.25 | MT010988 | Ex-type |
|  | CBS 201.37 | MW402112 |  |
|  | NY 001.E9 | MN534034 |  |
| *Fusarium proliferantum* | CBS 480.96 | MN534059 | Epitype |
|  | CBS 217.76 | AF160280 |  |
|  | CPC 39666 | PP764740 |  |
|  | F026 | MZ399213 |  |
| *Fusarium fujikuroi* | CBS 221.76 T | AB725605 | Ex-type |
|  | CBS 257.52 | MW402119 |  |
|  | CBS 240.64 | MW402117 |  |
| *Fusarium incarnatum* | CBS 132.73 | MN170476 | Ex-type |
|  | CBS 132907 | MN170477 |  |
|  | CBS 130314 | GQ505615 |  |
| *Fusarium oxysporum* | CBS 144134 | MH485044 | Ex-epitype |
|  | CBS 221.49 | MH484963.1 |  |
|  | JW 231014 | MZ921882 |  |

Note: *Fusarium* strains of the present study are marked in bold.

**Supplementary Table 2: Correlation matrix (Pearson) between germination-linked traits and biochemical compounds**

| **Variables** | **GP** | **RL** | **SL** | **WSL** | **SV** | **NoR** | **R_DPPH** | **S_DPPH** | **CR** | **CS** | **R_H2O2** | **S_H2O2** | **R_MDA** | **S_MDA** | **RFW** | **SFW** | **RDW** | **SDW** |
| --- | --- | --- | --- | --- | --- | --- | --- | --- | --- | --- | --- | --- | --- | --- | --- | --- | --- | --- |
| Germination % | 1 |  |  |  |  |  |  |  |  |  |  |  |  |  |  |  |  |  |
| Root length | 0.77 | 1 |  |  |  |  |  |  |  |  |  |  |  |  |  |  |  |  |
| Shoot length | 0.65 | 0.94 | 1 |  |  |  |  |  |  |  |  |  |  |  |  |  |  |  |
| WSL | 0.76 | 1.00 | 0.96 | 1 |  |  |  |  |  |  |  |  |  |  |  |  |  |  |
| Seedling vigour | 0.89 | 0.97 | 0.89 | 0.97 | 1 |  |  |  |  |  |  |  |  |  |  |  |  |  |
| Number of roots | 0.70 | 0.69 | 0.77 | 0.72 | 0.73 | 1 |  |  |  |  |  |  |  |  |  |  |  |  |
| Root DPPH (%) | -0.77 | -0.78 | -0.65 | -0.76 | -0.81 | -0.51 | 1 |  |  |  |  |  |  |  |  |  |  |  |
| Shoot DPPH (%) | -0.85 | -0.45 | -0.24 | -0.41 | -0.61 | -0.39 | 0.76 | 1 |  |  |  |  |  |  |  |  |  |  |
| Root carotenoid | -0.70 | -0.14 | 0.06 | -0.10 | -0.35 | -0.23 | 0.42 | 0.90 | 1 |  |  |  |  |  |  |  |  |  |
| Shoot carotenoid | -0.94 | -0.90 | -0.76 | -0.88 | -0.96 | -0.66 | 0.90 | 0.79 | 0.54 | 1 |  |  |  |  |  |  |  |  |
| Root H2O2 | -0.58 | 0.04 | 0.22 | 0.07 | -0.18 | -0.12 | 0.24 | 0.80 | 0.98 | 0.37 | 1 |  |  |  |  |  |  |  |
| Shoot H2O2 | -0.87 | -0.67 | -0.48 | -0.63 | -0.77 | -0.46 | 0.93 | 0.94 | 0.71 | 0.91 | 0.56 | 1 |  |  |  |  |  |  |
| Root MDA | -0.56 | 0.05 | 0.23 | 0.09 | -0.17 | -0.11 | 0.22 | 0.79 | 0.98 | 0.35 | 1.00 | 0.54 | 1 |  |  |  |  |  |
| Shoot MDA | -0.20 | -0.10 | 0.04 | -0.07 | -0.12 | 0.09 | 0.66 | 0.57 | 0.31 | 0.33 | 0.22 | 0.64 | 0.20 | 1 |  |  |  |  |
| Root fresh weight | 0.94 | 0.75 | 0.62 | 0.74 | 0.86 | 0.69 | -0.69 | -0.78 | -0.64 | -0.89 | -0.52 | -0.80 | -0.50 | -0.12 | 1 |  |  |  |
| Shoot fresh Weight | 0.62 | 0.51 | 0.38 | 0.49 | 0.59 | 0.18 | -0.74 | -0.72 | -0.55 | -0.69 | -0.44 | -0.76 | -0.43 | -0.53 | 0.48 | 1 |  |  |
| Root dry weight | 0.92 | 0.72 | 0.57 | 0.7 | 0.84 | 0.61 | -0.66 | -0.79 | -0.71 | -0.87 | -0.59 | -0.78 | -0.58 | -0.10 | 0.87 | 0.64 | 1 |  |
| Shoot dry weight | 0.78 | 0.64 | 0.51 | 0.62 | 0.74 | 0.42 | -0.59 | -0.66 | -0.57 | -0.79 | -0.47 | -0.70 | -0.46 | -0.12 | 0.68 | 0.54 | 0.74 | 1 |

Values in bold are different from 0 with a significance level alpha = 0.05. **GP**; Germination %, **RL**; Root length, **SL**; Shoot length, **WSL**; Whole seedling length, **SV**; Seeding vigour, **NoR**; Number of roots, **R_DPPH;** Root DPPH content, **S_DPPH**; Shoot DPPH content, **CR**; Root carotenoid content, **CS**; Shoot carotenoid content, **R_MDA**; Root Malondialdehyde content, **S_MDA**; Shoot Malondialdehyde content.
